# From aid dependency to market transformation: harnessing international trade and innovative business models to revitalize the assistive technology ecosystem in Africa

**DOI:** 10.3389/fresc.2026.1905487

**Published:** 2026-09-17

**Authors:** Mendemwelabou Timothée Pakouyowou

**Affiliations:** African Federation of Rehabilitation Professionals (FATO), International Project Coordinator, UNIDO Sub-Regional Office for Nigeria and ECOWAS, Abuja, Nigeria

**Keywords:** Africa, African Continental free Trade Area, assistive technology, disability inclusion, humanitarian development nexus, international trade, market development, prosthetics and orthotics

## Abstract

Africa faces a widening and largely unaddressed gap in access to assistive technology (AT). More than 240 million people on the continent require at least one assistive product, yet fewer than 3% of those in sub-Saharan Africa currently have access. The principal causes are four interlocking structural market failures: restrictive trade policies that inflate import costs, chronically underdeveloped local manufacturing capacity, inadequate national health financing for rehabilitation, and the absence of sustainable business models capable of maintaining AT services beyond the lifespan of time-limited humanitarian projects. These failures manifest with markedly different severity across the continent's 54 countries; this paper does not treat Africa as a uniform policy environment and distinguishes throughout between stable lower-middle-income contexts and fragile or conflict-affected states. This paper draws on more than 18 years of direct field experience in humanitarian and development programming across Ethiopia, the Democratic Republic of Congo, Mali, Algeria, Niger, Ukraine, and Nigeria, combined with a non-systematic review of peer-reviewed literature and institutional reports published between 2015 and 2024. Four interconnected policy solutions are proposed and analysed: (i) tariff reform and regulatory harmonisation under the African Continental Free Trade Area; (ii) expansion of local prosthetic and orthotic manufacturing capacity through diversified technology transfer, encompassing South South, triangular, and North South cooperation modalities; (iii) development of sustainable service delivery and financing models, including social enterprise with tiered cross-subsidy, output-based public private partnerships, and social health insurance; and (iv) systematic demand financing through national insurance benefit expansion and results-based financing mechanisms. The paper concludes with differentiated, time-horizoned recommendations short-term, medium-term, and long-term addressed to four distinct stakeholder groups: African governments and the African Union, development organisations and donors, the private sector, and the humanitarian community, with explicit acknowledgement that feasibility varies sharply by country context. The central argument is that durable access to AT in Africa requires deliberate, context-adapted market architecture, not simply expanded humanitarian aid.

## 1 Introduction

At the 2019 World Congress of the International Society for Prosthetics and Orthotics (ISPO) held in Kobe, Japan, an African rehabilitation delegate recounted a situation familiar to practitioners across the continent: a patient had travelled two days to reach a prosthetics clinic, only to discover that the required components were out of stock and would not arrive for a further three months, after an initial wait of eight months. The patient returned home without care. This account encapsulates a systemic failure that neither individual goodwill nor incremental increases in humanitarian donations can resolve. It points instead to the absence of the market architecture trade policy, regulatory frameworks, financing systems, and local production capacity required to make assistive technology reliably and sustainably accessible.

Assistive technology encompasses a broad spectrum of devices and systems, including prostheses, orthoses, wheelchairs, hearing aids, visual aids, and augmentative and alternative communication devices, that are essential for persons with disabilities to participate fully in society, live independently, and maintain their dignity. According to the World Health Organization (WHO), more than 2.5 billion people worldwide will require at least one assistive product by 2050; currently, only 5 to 15% of those in need in low- and middle-income countries (LMICs) have access (1). In sub-Saharan Africa, this proportion falls below 3% (2).

The humanitarian dimensions of this access deficit are acute and worsening. Survivors of anti-personnel landmines and explosive remnants of war concentrated in Angola, Mozambique, Somalia, South Sudan, Nigeria, and the Sahel experience amputation rates substantially above the global average (3). The International Campaign to Ban Landmines (ICBL) recorded 5,757 casualties in 2023, the highest figure in over two decades, with more than 85% occurring in Africa, the Middle East, and Asia (4). Extensive field experience with the International Committee of the Red Cross (ICRC) in Mali, as the WHO rehabilitation focal point in the Democratic Republic of Congo (DRC) and Niger, and through the African Federation of Rehabilitation Professionals (FATO) continental network consistently identifies the absence of functional AT systems rather than deficits of clinical skill, political will, or expressed need as the binding constraint on rehabilitation outcomes in these contexts.

## 2 Methodology and analytical approach

This paper argues that the AT access gap in Africa is principally a structural market problem requiring simultaneous and coordinated reform across trade, production, financing, and regulatory systems, not simply expanded humanitarian aid. Critically, however, the nature, sequencing, and feasibility of this reform differ substantially across country contexts; a recommendation that is immediately actionable in a stable, growing economy may be inapplicable in a state experiencing active armed conflict. This heterogeneity is addressed explicitly throughout the paper, and most directly in Section 3.3. The analysis draws on primary evidence from United Nations Industrial Development Organization (UNIDO) Project 240280 in Nigeria a technology transfer initiative introducing 3D-printed prosthetics alongside extensive practitioner field evidence and key normative documents, including the WHO Global Report on Assistive Technology (1), the United Nations Convention on the Rights of Persons with Disabilities (CRPD) (5), and the African Union (AU) Protocol on the Rights of Persons with Disabilities in Africa (6). Comparative policy insights are systematically integrated from the Indian experience documented by Prajapati et al. (7), who analyse complementary challenges in prosthetic and orthotic service delivery, workforce development, and system-level reform in a lower-middle-income context, and whose proposed solutions including government-scale financing schemes, innovation ecosystem development, logistics modernisation, and public awareness campaigns carry direct relevance for African settings.

The paper is organised into nine sections covering methodology, structural diagnosis, trade-based and business model solutions, the humanitarian market nexus, policy recommendations, a terminology reference, and a conclusion.

This study is a practitioner-informed policy analysis that triangulates three evidence streams: direct field observations, peer-reviewed literature, and grey and institutional documentation. It does not employ a formal systematic review methodology and does not claim the reproducibility of Preferred Reporting Items for Systematic Reviews and Meta-Analyses (PRISMA)-compliant reviews. The objective is analytical interpretation of structural barriers and enabling conditions for sustainable AT market development in Africa, with the aim of generating actionable, evidence-grounded policy guidance for practitioners and decision-makers.

### 2.1 Practitioner evidence

The primary evidence base comprises more than 18 years of direct field experience in rehabilitation and AT programming across more than ten countries. Key operational contexts include conflict-affected and post-conflict settings in Mali, the DRC, Niger, Algeria, and South Sudan through Humanity & Inclusion (HI) and the ICRC, and public health system strengthening in Ethiopia and Nigeria through WHO and UNIDO. Technology transfer programme coordination under UNIDO Project 240280 (2023 2026) constitutes the principal case study, encompassing equipment installation at four institutional sites: the Medical Rehabilitation Therapists Board (MRTB) headquarters in Abuja, the National Orthopaedic Hospital Dala Kano (NOHDK), the University Teaching Hospital Maiduguri, and the Federal University of Technology Owerri (FUTO). The first author's direct institutional role as Project Coordinator is explicitly acknowledged as a potential source of confirmation bias; the reader is encouraged to weigh this when evaluating case-specific findings and recommendations.

### 2.2 Literature review

A non-systematic review of peer-reviewed and grey literature was conducted, covering publications from 2015 to 2024. Search terms included “assistive technology Africa,” “prosthetics and orthotics market low-income countries,” “3D printing prosthetics,” “AT financing,” “disability trade policy,” “AfCFTA health,” “public private partnerships rehabilitation,” and related variants. Sources were drawn from PubMed, Google Scholar, WHO institutional repositories, ATscale publications, and World Bank databases. The review acknowledges three important evidential constraints: few randomised controlled studies exist on AT business models in low-income settings; cost data for 3D-printed prostheses are highly context-dependent; and published case studies of public private partnership (PPP) models in AT predominantly document positive outcomes, suggesting substantial publication bias. Where evidence is weak, contested, or absent, this is stated explicitly in the relevant sections.

### 2.3 Grey literature and institutional documents

Two internal UNIDO Nigeria documents are cited: a monthly project progress report (8) and a technical evaluation report from the procurement process (9). Both documents are identified as grey literature, used exclusively to document the operational context of Project 240280, and have not undergone external peer review. Consistent with UNIDO's institutional confidentiality obligations for internal project and procurement records, these documents cannot be released as supplementary materials; they remain available for verification by the journal's editorial team on a confidential basis upon request, and the corresponding author can facilitate such requests through UNIDO's communications office. The IfeanHealth commercial bid submission (10) is similarly a confidential procurement document, cited solely to illustrate a specific business model configuration; no performance claims are advanced on its basis, and no proprietary commercial data from the submission are disclosed in this paper.

### 2.4 Limitations

Five principal limitations are acknowledged and reflected throughout the analysis. First, and most importantly, this paper's use of “Africa” as an analytical frame should not be read as treating the continent as a single market or policy environment. The 54 countries of Africa differ profoundly in economic development, health financing architecture, regulatory capacity, manufacturing infrastructure, and rehabilitation workforce density. The country cases most frequently cited Kenya, Rwanda, Togo, Uganda, Tanzania, and Nigeria are predominantly stable or stabilising lower-middle-income states; they are not representative of the continent as a whole, and several of the recommendations developed in Section 7 presuppose a baseline of functioning state institutions, predictable customs administration, and physical access to national territory that does not hold in every African country. Section 3.3 develops this heterogeneity explicitly, with particular attention to fragile and conflict-affected states such as Somalia, Mali, Niger, and the DRC, where active armed conflict, including jihadist insurgency in the Sahel and eastern DRC, substantially constrains both border control and policy implementation capacity. Cases of failed technology transfer or collapsed PPP arrangements are, in addition, substantially underrepresented in the accessible literature, which tends to document successful interventions (11). Second, the cost comparison between approximately USD 200 for 3D-printed prostheses and USD 1,500 5,000 for conventionally fabricated equivalents reflects direct material and printing costs only; equipment amortisation, electricity, technician time, clinical follow-up, device longevity, and patient travel costs are excluded, and a full lifecycle cost-effectiveness assessment has not been conducted (12). Third, clinical evidence on the durability and long-term functional outcomes of 3D-printed prostheses remains limited; this paper does not assert clinical equivalence across patient profiles, amputation levels, or functional demand categories (13). Fourth, since equipment installation under UNIDO Project 240280 was completed in May 2026, concurrent with the preparation of this article, long-term operational sustainability cannot yet be demonstrated and the project should be understood as an early pilot, not a proven model. Fifth, several arguments in this paper draw on internal UNIDO and commercial grey literature that cannot be independently verified by readers; as noted in Section 2.3, these sources are identified transparently as such, and the claims they support are stated with corresponding caution.

## 3 The scale and structural roots of the assistive technology access gap in Africa

### 3.1 Epidemiology and magnitude of unmet need

Africa accounts for approximately 17% of the global population yet bears a disproportionately high burden of disability from road traffic injuries, conflict-related trauma, anti-personnel landmines, and preventable conditions including poliomyelitis sequelae and lower-limb complications of diabetes mellitus (14). In Nigeria the continent's most populous country and the primary field site of this analysis an estimated 1.7 million people live with limb loss or limb difference, yet there are fewer than 0.05 prosthetic and orthotic (P&O) professionals per 100,000 population, far below the WHO-recommended minimum of three per 100,000 (1, 15). At the National Orthopaedic Hospital Dala Kano (NOHDK), direct field observation during UNIDO Project 240280 confirmed that patients routinely wait multiple months for prosthetic fittings, with chronic component shortages systematically compounding delays. This pattern reflects not a marginal resource constraint but a deep, systemic supply failure that requires structural rather than incremental remediation.

The access gap is most severe in conflict-affected and fragile states, in rural and peri-urban areas, and among women and girls, who face compounding barriers including restricted mobility, social stigma, economic marginalisation, and limited health literacy that impedes navigation of formal service systems (16). Field observations across Ethiopia, the DRC, Mali, Niger, and Algeria over nearly two decades reveal a self-reinforcing cycle: conflict and fragility generate disability; disability generates poverty; and poverty prevents acquisition of the AT devices that could restore economic participation and social inclusion. Humanitarian aid can interrupt this cycle temporarily; only functional, self-sustaining market systems can sustain the interruption over the multi-decadal timeframes required for genuine structural transformation.

### 3.2 A four-dimension market failure framework

Health economics analysis identifies four interlocking market failure mechanisms that collectively account for the severity and persistence of the AT access gap in Africa (11). Each mechanism is self-reinforcing and intersects with the others; resolving any single dimension in isolation typically produces limited and transient gains. Understanding this systemic interdependence is essential to designing interventions that address structural causes rather than proximate symptoms.

The first and foundational failure is information asymmetry. Users, caregivers, clinical prescribers, and policymakers commonly lack reliable knowledge of available AT products, their technical specifications, functional outcomes, and comparative costs. This suppresses both demand articulation and competitive market entry, perpetuating dependence on a narrow set of imported products whose characteristics and pricing are controlled by suppliers outside Africa. Practical countermeasures include commissioning national AT needs assessments using standardised disability prevalence metrics; developing open-access product catalogues aligned with the WHO Priority Assistive Products List (APL); deploying community-facing awareness and education platforms that combine mobile health applications, community radio, and peer ambassador networks of experienced AT users; and systematically publishing the market intelligence generated by humanitarian procurement operations supplier registers, price benchmarks, quality data, and geographic service maps as freely accessible public goods.

The second failure is supply-side inadequacy. Local manufacturing and assembly capacity for AT products is minimal across Africa; supply chains are fragmented, predominantly import-dependent, and highly vulnerable to currency fluctuations, logistics failures, and regulatory inconsistencies at borders. Specialised AT distributors are largely absent outside major capital cities, producing severe last-mile access deficits in rural and peri-urban areas. Addressing this failure requires coordinated investment in local manufacturing including 3D additive manufacturing as a structural cost-reduction pathway alongside targeted procurement reform, structured P&O workforce training, and systematic distribution system development, as elaborated in Section 4.

The third failure is demand-side suppression. Chronically low household purchasing power, the near-universal absence of AT coverage in national health insurance benefit packages, and systematic underfunding of rehabilitation services collectively prevent effective demand from reaching levels that would attract commercial investment or justify local production at commercially viable scale (17, 18). Insurance integration and results-based financing mechanisms, examined in detail in Section 5, represent the primary structural responses to this failure dimension.

The fourth failure is regulatory and trade barriers. Import tariffs on AT components range from 5% to 30% across 18 African countries surveyed by ATscale (19). Inconsistent national product safety and clinical quality standards, combined with the absence of harmonised continental procurement frameworks, inflate landed costs, deter regional market integration, and substantially complicate quality assurance for locally manufactured devices. Regulatory reform including the proposed African Continental Free Trade Area (AfCFTA) Assistive Products Schedule is the subject of Section 4.1.

These four failures are mutually reinforcing: elevated import costs restrict supply; weak supply limits access; constrained access obscures true demand; and depressed, invisible demand reduces the political salience of regulatory and trade reform (11). Breaking this cycle requires simultaneous, coordinated action across all four dimensions simultaneously. This systemic interdependence justifies, and indeed necessitates, the multi-pillar analytical and policy framework developed in the sections that follow while the country-context heterogeneity discussed in Section 3.3 determines how, and how quickly, that framework can realistically be applied in any given setting.

### 3.3 Country heterogeneity: why “Africa” is not one market

The preceding analysis, like much of the AT literature, necessarily speaks of structural failures “across Africa.” This framing carries an analytical risk that must be made explicit: the continent comprises 54 sovereign states with vastly different economic structures, regulatory capacities, health financing systems, and security conditions. A policy lever that is immediately actionable in one country may be entirely infeasible in another, and the difference is rarely one of degree alone.

At one end of this spectrum sit countries such as Kenya, Rwanda, South Africa, and with important qualifications Nigeria, which combine functioning customs administrations, growing manufacturing bases, and the institutional continuity needed to negotiate and implement tariff reform, insurance benefit expansion, or PPP contracting over multi-year horizons. The zero-tariff regimes documented in Kenya and Rwanda (20), for example, presuppose a level of regulatory predictability and enforcement capacity that cannot be assumed everywhere.

At the other end sit states experiencing active armed conflict or chronic fragility, where the preconditions for most of this paper's recommendations are partially or wholly absent. In *Somalia*, *Mali*, and *Niger*, protracted jihadist insurgency has degraded state authority over substantial portions of national territory, disrupting customs administration, health ministry oversight, and the continuity of professional certification systems that AfCFTA tariff reform, national AT coordination units, and PPP contracting all depend upon. In the *Democratic Republic of the Congo*, the practical difficulty of controlling porous and contested eastern borders undermines tariff enforcement and creates parallel informal supply chains that a formal Assistive Products Schedule cannot easily reach. In each of these contexts, government capacity to implement national policy is itself contested territory a condition fundamentally different from the implementation challenges, however serious, faced by a stable lower-middle-income state.

This does not mean that structural market-building is irrelevant in fragile and conflict-affected states; rather, it means that the sequencing, instruments, and expected pace of reform must be adapted. In these contexts, humanitarian actors with established field presence and community trust such as the ICRC and Humanity & Inclusion are likely to remain the primary, and in places the only, deliverers of AT services for the foreseeable future, and the market-building agenda articulated in Sections 4 through 6 should be understood as a medium-to-long-term horizon for these states, pursued opportunistically as security and governance conditions permit, rather than as an immediately implementable programme. Recommendations throughout Section 7 are therefore explicitly tagged by the country-context category for which they are most directly applicable: stable and stabilising states; lower-middle-income states with moderate institutional capacity; and fragile or conflict-affected states.

## 4 International trade as a lever for assistive technology market transformation

### 4.1 Tariff reform and the AfCFTA opportunity

The African Continental Free Trade Area (AfCFTA), formally launched in January 2021, offers an unprecedented structural opportunity to reshape trade conditions for assistive products across 54 participating member states (21). This paper proposes the creation of a dedicated Assistive Products Schedule within the AfCFTA goods protocol, modelled on the precedent of the WHO Model List of Essential Medicines. Such a schedule would establish zero intra-African tariffs on AT products, reduce tariffs on imported components from non-African sources, set binding shared quality and safety standards applicable to all products traded under the schedule, and create a continental preferential procurement framework that systematically advantages locally manufactured devices over equivalent imports. This instrument is, by its nature, most readily implementable among states with functioning customs administrations and regulatory institutions; its application in fragile or conflict-affected states, discussed in Section 3.3, will necessarily lag and may require complementary humanitarian-channel exemptions.

The political feasibility of this approach has already been demonstrated at national level in stable institutional contexts. Kenya and Rwanda have independently implemented zero-tariff regimes on defined ranges of assistive products, with documented improvements in product availability and measurable reductions in retail prices (20). International Finance Corporation (IFC) modelling projects that a continental-level tariff reduction through the AfCFTA could reduce landed AT product costs by 15 25%, though these projections depend on modelling assumptions that warrant independent empirical validation (22). FATO has advocated directly with the AU Commission for this reform; the reader should weigh this institutional position when evaluating the recommendation's independence.

Regulatory harmonisation is an equally important, if less visible, component of trade barrier reduction. Inconsistent national certification standards for prostheses and orthoses which are distinct product and professional practice categories (see Section 8) constitute non-tariff barriers that impede regional market integration and substantially complicate cross-border technology transfer. Three specific regulatory measures should be prioritised: (i) developing harmonised device quality standards for prostheses and orthoses within the African Organisation for Standardisation (ARSO) framework, aligned with International Organization for Standardization (ISO) standards where applicable; (ii) establishing mutual recognition agreements among national P&O professional certification bodies to enable qualified practitioners to work across borders, expanding effective workforce capacity without increasing training costs; and (iii) creating a continental adverse event reporting system for AT devices that generates post-market surveillance data currently entirely absent across the African region.

### 4.2 Technology transfer: expanding beyond south south models

South South and Triangular Cooperation (SSTC) has emerged as one of the most operationally tested mechanisms for expanding AT production capacity in Africa (23). UNIDO Project 240280 facilitating the transfer of Japanese 3D scanning, computer-aided design (CAD), and additive manufacturing technology to Nigerian P&O professionals through a partnership between UNIDO, Instalimb Inc., and four Nigerian institutional sites serves as the primary case examined in this paper. The project demonstrated that a custom-fitted prosthetic limb can be fabricated in under 60 min using digital workflows, at direct material and printing costs estimated at approximately USD 200 per device, compared to USD 1,500 5,000 for conventionally fabricated equivalents. As noted in Section 2.4, a full lifecycle cost-effectiveness analysis incorporating equipment amortisation, training, clinical follow-up costs, and device longevity has not been conducted and is required before these figures are used for policy benchmarking (12). Equipment was handed over to NOHDK management in May 2026; long-term operational sustainability remains to be demonstrated (8).

Critically, technology transfer for AT market development cannot be confined to South South channels. The Indian experience documented by Prajapati et al. (7) illustrates four complementary modalities that African programme designers and policymakers should systematically evaluate. (i) government-administered demand-side subsidy schemes exemplified by India's Assistance to Disabled Persons (ADIP) programme have scaled AT device acquisition to large, geographically dispersed populations at low unit cost by pooling government purchasing power and tendering across multiple competing suppliers. (ii) start-up and innovation ecosystems developed through structured public private collaboration have produced AT solutions adapted to local anthropometric standards, climatic conditions, and available materials at costs substantially below those of imported equivalents. (iii) investment in modern logistics infrastructure including last-mile distribution networks, digital inventory management systems, and decentralised device repair hubs has demonstrated that supply chain modernisation can reduce AT access failures substantially and independently of manufacturing cost reductions. (iv) structured multi-channel public awareness campaigns, supported by peer networks of AT users as credible community ambassadors, have demonstrably expanded informed demand and reduced stigma-driven suppression of device acquisition across diverse low-income settings. Each of these modalities is directly transferable to African contexts with adequate institutional baseline capacity, and should be incorporated into future technology transfer and AT market development programming.

Triangular cooperation arrangements in which a high-income donor funds a middle-income partner with demonstrated AT capacity to transfer knowledge and technology to lower-income African recipients combine the contextual sensitivity of South South exchange with the resources of North South cooperation. Three structural conditions are necessary for any technology transfer modality to generate lasting AT market development rather than temporary demonstration effects: (i) genuine adaptation of the technology to the local skills base, available materials, and clinical environment, rather than direct transplantation of high-income country solutions; (ii) parallel development of the regulatory framework, including P&O professional certification through national bodies such as Nigeria's MRTB, quality assurance standards for 3D-fabricated devices, and insurance reimbursement pathways; and (iii) demonstrated institutional and financial capacity in the receiving organisation to sustain operations independently after the external project cycle ends (8). Without all three conditions in place conditions that are themselves a function of the institutional stability discussed in Section 3.3 technology transfer produces capability without continuity.

### 4.3 Public procurement as a market-shaping instrument

Public procurement represents 10 30% of gross domestic product (GDP) in most African countries and constitutes a systematically underutilised instrument for AT market development (24). Strategically deployed, government purchasing power can anchor demand certainty for local producers, reducing the investment risk that currently deters entry; establish quality benchmarks that progressively raise sector standards; compress unit costs through volume aggregation; and generate market intelligence supplier capacity data, competitive price ranges, and product quality assessments that otherwise exists only in fragmented, inaccessible form.

The UNIDO Nigeria competitive tendering process for P&O components under Project 240280 illustrates this market-shaping function directly. The process engaged five vendors from four countries, including two Nigerian enterprises, and produced competitive pricing, documented market intelligence, and a formal supplier register for prosthetic and orthotic components a structured, publicly accessible asset that had not previously existed in Nigeria in any systematic form (9). Both the World Bank and the WHO Global Cooperation on Assistive Technology (GATE) initiative identify procurement reform as a priority lever for AT market development in low-income settings (24, 25).

Three practical procurement reform measures should be implemented. (i) mandatory local content requirements of 20 30% in AT tenders, applied progressively as national manufacturing capacity develops, would channel government purchasing power toward domestic producers and stimulate local value chain investment. (ii) government framework agreements with pre-qualified regional AT suppliers would substantially reduce transaction costs for smaller institutional procurers who currently lack the administrative capacity to manage independent competitive processes. (iii) mandatory public disclosure of AT procurement data prices paid, quantities, supplier identities, and quality outcomes would systematically reduce information asymmetries for both buyers and potential market entrants, directly addressing the first of the four core market failures identified in Section 3.2. All three measures presuppose a baseline procurement administration capacity that, as discussed in Section 3.3, is unevenly distributed across the continent.

## 5 Sustainable business model innovation for the assistive technology sector in Africa

### 5.1 The structural limits of the humanitarian donation model

In low-income African settings, the dominant modality for AT provision remains humanitarian donation: non-governmental organisations and charities supply prostheses and wheelchairs that are predominantly imported, frequently poorly matched to local clinical needs and user anthropometry, and entirely dependent on external funding cycles. This model does not merely fail to develop markets; it actively suppresses them. When donated products arrive, local providers serving paying clients cannot compete on price and are forced to exit; when donations subsequently cease as they inevitably do at the close of every funding cycle no viable local providers remain to serve the population. Field observations with HI and the ICRC across West and Central Africa over fifteen years document this pattern repeatedly. The Lancet Commission on Global Health and Disability estimates that fewer than 2% of AT provision in sub-Saharan Africa is delivered through commercially sustainable models; the remaining 98% depends on humanitarian funding or catastrophic out-of-pocket expenditure, neither of which constitutes a durable or equitable solution (17). Comparable user-level evidence from Bangladesh a lower-middle-income context with structural similarities to several African settings documents the same predictable institutional cycle: elevated expectations during humanitarian programming phases, followed by service collapse when external funding ends (26). Meaningful and lasting progress requires a fundamental shift in the underlying financing and delivery paradigm; this shift, however, is realistically available only where a minimum threshold of market and institutional stability already exists, a point developed further in Section 5.2 and Section 7.

### 5.2 Four pathways toward sustainable AT business models

Drawing on field evidence across multiple African countries and systematic analysis of AT enterprises in East, West, and Southern Africa, four business model pathways toward sustainability are identified and analysed below. These pathways are not mutually exclusive; resilient AT service ecosystems will typically combine elements of several simultaneously. They are also not uniformly available: all four presuppose, to varying degrees, a baseline of market demand, institutional continuity, and regulatory predictability that is unevenly distributed across the country contexts discussed in Section 3.3, and they are, accordingly, considerably more readily implementable in stable lower-middle-income states than in fragile or conflict-affected ones. Each pathway also maps onto the short-, medium-, and long-term implementation timeline presented in Section 7.

#### 5.2.1 Pathway 1: social enterprise with tiered cross-subsidy

AT providers in larger urban centres can serve insured patients and institutionally contracted clients such as government agencies and military hospitals at cost-reflective market rates, deploying the resulting operating surplus to cross-subsidise services for uninsured and economically marginalised populations, including landmine survivors and conflict-affected amputees. The IfeanHealth model in Nigeria illustrates this configuration: an official Ottobock original equipment manufacturer (OEM) partnership combined with institutional supply contracts for the Nigerian Army and national orthopaedic hospitals generates a commercial revenue base, while corporate social responsibility (CSR)-funded community outreach extends coverage to indigent patients who would otherwise be excluded (10). The model's replicability is structurally constrained by the requirement for a paying market of sufficient size and density to generate adequate cross-subsidy surplus, which limits direct applicability in very low-income, sparsely populated, or insecure contexts. Development finance institutions (DFIs) should catalyse this pathway through concessional loans, equity participation, and first-loss guarantee instruments targeted specifically at AT social enterprises with explicit blended-value mandates and measurable social impact obligations.

#### 5.2.2 Pathway 2: output-based public private partnerships

Output-based aid arrangements, in which governments or development donors contract accredited private AT providers and disburse payment per independently verified service outcome, have been piloted in Uganda and Tanzania, with reported improvements in both service quality and cost efficiency relative to direct government provision (27). These PPP models allocate delivery risk more efficiently than input-based contracting: providers bear the operational risk of service delivery, while purchasers bear only the risk of outcome verification. Their success depends critically, however, on three preconditions that are frequently absent in low-resource environments, and almost universally absent in fragile or conflict-affected ones: robust and affordable outcome verification systems; an accreditation framework genuinely achievable by private providers operating with limited capital; and sufficient government administrative capacity to design and sustain contracting relationships over multi-year periods. Where these preconditions have not been met, PPP models have underperformed or collapsed entirely a dimension of the evidence substantially underrepresented in the positively biased published literature. Development partners should provide dedicated technical assistance for PPP design and provider accreditation, while DFIs deploy first-loss guarantee facilities to attract private capital during the high-risk early phases of PPP establishment.

#### 5.2.3 Pathway 3: social health insurance and results-based financing

Togo's national health insurance architecture provides the most directly instructive African precedent for insurance-based AT access financing. The Institut National d'Assurance Maladie (INAM), covering civil servants, and the Assurance Maladie Universelle (AMU), serving the broader population, together include basic AT coverage without supplementary payment at the point of service (18). This demonstrates that insurance-based AT access is administratively and fiscally feasible in a West African lower-middle-income context, not merely in higher-income settings though Togo, notably, also benefits from comparatively stable governance and territorial control, conditions that materially enabled this reform. Ghana's National Health Insurance Scheme and Kenya's Social Health Authority provide comparable, if higher-income, precedents (28). The Togo model should be systematically presented by the Economic Community of West African States (ECOWAS) and the African Union to member states as a replicable template, accompanied by targeted technical assistance for AT benefit package costing and provider reimbursement rate-setting, recognising that the pace of adoption will necessarily differ by national context.

Results-based financing (RBF) mechanisms in which donors and governments pay per verified AT service outcome rather than per input or activity completed offer a structurally complementary instrument that aligns provider incentives with patient outcomes across the value chain (27). RBF can be applied simultaneously at multiple system levels: disbursing to AT providers per successfully fitted and clinically assessed device; reimbursing P&O training institutions per certified graduate who enters and remains in active clinical practice; and providing performance-linked budget support to governments per percentage point increase in population coverage for AT under national insurance schemes. The systematic integration of RBF into both donor programming and national health financing architecture represents a significant and under-exploited structural innovation for the sector, most readily applicable where a functioning verification infrastructure already exists.

#### 5.2.4 Pathway 4: 3D additive manufacturing for structural cost reduction

The integration of 3D scanning, CAD, and additive manufacturing into prosthetics production represents the most operationally tested structural cost-reduction pathway currently available for the African context. A 2017 scoping review of assistive technology research in resource-limited environments confirms that prosthetics and mobility devices are among the most extensively documented AT categories in low- and middle-income settings, while also identifying substantial evidence gaps in system-level service delivery research relative to product-focused studies (13); long-term load-bearing durability of 3D-fabricated devices under conditions of intensive daily use remains a significant knowledge gap requiring dedicated prospective research. Rashid et al. (29) and Awad et al. (30) provide complementary scoping review and clinical evidence on manufacturing quality and patient-reported outcomes across low-resource settings.

Scale-up of 3D prosthetics manufacturing in Africa requires three enabling conditions to be developed concurrently. (i) local and regional availability of printing consumables particularly suitable thermoplastic filaments with approved mechanical properties at commercially viable prices must be secured; dependence on imported consumables partially offsets the unit cost advantages of local fabrication. Second, national P&O certification bodies must develop, consult on, and publish regulatory frameworks for the clinical recognition of 3D-fabricated prostheses, including approved material specifications and quality assurance protocols, to enable reimbursement by insurance schemes. (iii) clinical protocols defining the patient profiles, amputation levels, and functional activity categories for which 3D fabrication is appropriate, and those for which conventional fabrication remains the clinical standard of care, must be developed and formally adopted by national and regional P&O professional associations. Without all three enabling conditions in place concurrently and without the underlying regulatory and institutional stability discussed in Section 3.3 3D manufacturing capacity will remain confined to externally funded demonstration projects with limited systemic impact.

## 6 The humanitarian market nexus: building systems, not services alone

The tension between immediate humanitarian service delivery and long-term system building is a well-documented structural challenge in international development (31). In AT, this tension is particularly acute for three distinct reasons. First, the products involved are technically specialised and expensive, making *ad hoc* procurement the default operational mode. Second, AT markets in most African contexts are absent or deeply dysfunctional, eliminating the commercial supply systems that other sectors can progressively engage. Third, humanitarian AT provision rarely transitions smoothly to commercial or government supply when project funding expires, because the market and regulatory conditions for such a transition have typically not been developed during the operational phase and in active conflict settings such as Somalia, Mali, Niger, and the eastern DRC, those conditions may not be developable at all until security conditions change. Longitudinal field experience in humanitarian AT programming in Mali (ICRC), Ethiopia (WHO), the DRC, Niger, Algeria, and Ukraine reveals a consistent institutional dynamic: operational performance pressures systematically prioritise immediate output metrics over systemic capacity investment (31). Procurement defaults to established international suppliers rather than local alternatives, which would require more intensive and uncertain supplier development processes. Training budgets are consistently the first casualty of funding compression. Regulatory engagement is treated as a government responsibility external to programme scope rather than a deliverable within it. The cumulative result is a sector structurally dependent on emergency funding for services that are, in stable contexts, in principle commercially viable and deliverable (32).

Three evidence-based institutional shifts are proposed for humanitarian AT programming, with the explicit caveat that their applicability is conditional on local security and governance conditions. First, procurement should be progressively localised where conditions permit. Humanitarian AT purchasing should be systematically channelled through local and regional suppliers wherever technical specifications can be met and supply routes are secure, generating supplier capacity and market intelligence as by-products of operational procurement rather than treating market development as a separate, post-programme activity. The ICRC Physical Rehabilitation Programme has advanced furthest in this direction among international humanitarian actors, including in several fragile settings, and provides an institutionally documented and replicable operational model (33).

Second, regulatory engagement should be embedded as a core, budgeted programme component where a functioning regulatory counterpart exists. P&O professional certification, quality assurance framework development, and insurance reimbursement pathway design should be treated as programme deliverables with dedicated budget lines and monitoring indicators, not as optional policy advocacy activities for which no resources are allocated. In UNIDO Project 240280, P&O training was deliberately structured within the MRTB certification framework, specifically to ensure that trained professionals could practice independently and commercially after project closure, thereby converting a project output into a durable, self-sustaining market contribution an approach that depended on Nigeria's functioning national regulatory infrastructure and would require substantial adaptation in a setting without such infrastructure.

Third, humanitarian organisations should systematically leverage their institutional positioning for market-enabling systemic advocacy where government counterparts retain functional authority. Organisations with sustained, trust-based government relationships are structurally better positioned than commercial actors to advocate for tariff reform, AT insurance inclusion, and regulatory framework development in post-conflict and fragile state contexts where some governance capacity persists. Translating sustained operational presence into targeted systemic advocacy remains the most underexploited potential contribution of the humanitarian sector to long-term AT market development (32). Bright et al. (34) and the WHO GATE initiative (25) both identify regulatory strengthening and P&O workforce development as necessary preconditions for sustainable AT market formation precisely the domains where humanitarian organisations hold institutional leverage but have historically under-invested relative to the scale of the opportunity, and precisely the domains that active conflict most directly undermines.

## 7 Policy recommendations

The following recommendations are derived directly from the evidence, analysis, and case material presented in this paper. They are organised by stakeholder group and across three implementation time horizons short-term (0 2 years), medium-term (3 5 years), and long-term (beyond 5 years) to enable practical planning by actors with different mandates, resource bases, and planning cycles. *Consistent with the heterogeneity discussed in* *Section 3.3, each recommendation is tagged with the country-context category for which it is most directly applicable:* **[Stable]** for stable or stabilising states with functioning institutions (e.g., Kenya, Rwanda, South Africa, Nigeria); **[LMIC]** for lower-middle-income states with moderate institutional capacity; and **[Fragile]** for fragile or conflict-affected states (e.g., Somalia, Mali, Niger, the DRC), where implementation will necessarily be slower, more partial, and more dependent on parallel governance and security gains. They are intended as evidence-informed starting points for policy design, not as prescriptive mandates, and must be adapted to specific national and institutional contexts.

### 7.1 For African governments and the African Union

#### 7.1.1 Short-Term actions (0 2 years)

- **Insurance inclusion** *[Stable/LMIC]:* Mandate the explicit inclusion of prostheses, orthoses, mobility devices, hearing aids, and visual aids in national health insurance benefit packages. Reimbursement rates must be set at levels that support viable provider operations, not merely symbolic inclusion. Countries with nascent insurance systems should begin with civil servant schemes as demonstrated by Togo's INAM model (18) and expand incrementally toward universal population coverage. This recommendation presupposes a functioning national insurance administration and is not readily applicable in states where territorial control is contested.
- **Immediate tariff removal** *[Stable/LMIC]:* Remove import duties unilaterally on all products included in the WHO Priority Assistive Products List as an immediately actionable, low-cost measure, pending the conclusion of AfCFTA negotiations. Kenya and Rwanda demonstrate that this is politically and administratively feasible within existing legal frameworks (20). In fragile states with limited border control, complementary measures such as duty waivers administered directly through verified humanitarian channels may be more realistic than economy-wide tariff reform.
- **National AT coordination** *[Stable/LMIC]:* Establish national AT coordination units within health ministry structures, integrating policy development, strategic procurement, quality assurance, and P&O workforce planning under a single institutional framework, using the WHO GATE model as the organising architecture (25).
- **National Rehabilitation Strategic Plans financing and implementation** *[Stable/LMIC]:* Prioritise the financing and full implementation of existing national rehabilitation strategic plans and disability-inclusive health sector strategies that incorporate AT provision and service delivery. Many African countries have already developed and ratified National Rehabilitation Strategic Plans including several supported by WHO and development partners which provide a ready-made policy framework for AT integration. Governments should embed AT service delivery targets within these plans, allocate dedicated domestic budget lines, and establish accountability mechanisms to track implementation progress against agreed indicators. Where such plans do not yet exist, their development should be treated as an immediate priority, using the WHO Rehabilitation 2030 framework and the GATE country capacity assessment tool as organising instruments (1, 25).

#### 7.1.2 Medium-term interventions (3 5 years)

- **AfCFTA Assistive Products Schedule** *[Stable/LMIC, with phased Fragile-state provisions]:* Negotiate and adopt a dedicated Assistive Products Schedule within the AfCFTA goods protocol, establishing zero intra-African tariffs, harmonised quality standards, and a continental preferential procurement framework, modelled on the WHO Model List of Essential Medicines approach (19, 21, 22). Implementation timelines and enforcement mechanisms should explicitly accommodate states where customs administration does not yet cover full national territory.
- **P&O Workforce Development Plans** *[Stable/LMIC]:* Develop national AT workforce plans aligned with WHO staffing benchmarks (minimum three P&O professionals per 100,000 population), incorporating accredited university-based and technician-level training curricula, regulatory recognition frameworks for 3D-fabricated prostheses and orthoses, and mandatory continuing professional development requirements (1, 15).
- **PPP piloting** *[Stable]:* Launch pilot output-based PPP contracts for AT services in at least two sub-national regions per country, with independent third-party outcome verification and structured evaluation against pre-defined impact indicators, using the Uganda and Tanzania models as operational references (27).

#### 7.1.3 Long-term policy and system reforms (beyond 5 years)

- **Universal AT coverage** *[Stable/LMIC]:* Achieve universal AT coverage within national health systems, integrating prosthetic and orthotic services into primary healthcare delivery platforms in rural and peri-urban areas through task-shifted, community-based P&O technicians working under national professional certification and supervision frameworks.
- **Continental regulatory authority** *[All contexts, long horizon]:* Establish a continental AT market regulatory body within the African Union framework, with a mandate for device safety standard-setting, mandatory adverse event reporting, and cross-border supplier accreditation analogous in function to the European Medicines Agency for pharmaceutical products in Europe.
- **Continental centre of excellence** *[All contexts, long horizon]:* Fund a continental AT innovation, research, and training centre connecting universities, technology transfer institutions, and private sector enterprises, mandated to develop Africa-adapted AT solutions and serve as the continent's primary hub for clinical and health economics research on 3D-fabricated prostheses and orthoses.

### 7.2 For development organisations and donors

#### 7.2.1 Short-term actions (0 2 years)

- **Redirect investment** *[Stable/LMIC]:* Progressively redirect programmatic investment from AT product donation toward local production capacity building, diversified technology transfer, P&O workforce training, and regulatory framework development, using UNIDO Project 240280 as a documented operational reference model (35).
- **Publish market intelligence** *[All contexts]:* Systematically document and publicly disseminate the market intelligence generated through humanitarian AT operations supplier registers, price benchmarks, quality assessment data, geographic service maps, and disaggregated needs assessments as a systematic contribution to reducing the information asymmetries that suppress commercial market entry (11).

#### 7.2.2 Medium-term interventions (3 5 years)

- **Diversify technology transfer** *[Stable/LMIC]:* Operationalise SSTC, triangular cooperation, and North South technology transfer mechanisms to deliver proven AT manufacturing and distribution technologies to African production hubs, encompassing both Japanese 3D prosthetics platforms and Indian government-scale demand subsidy models, with national P&O professional bodies as anchor institutions for certification and quality assurance (7, 23, 35).
- **Market development mandates** *[Stable/LMIC]:* Require explicit, measurable market development objectives in all AT programming: local supplier development targets, PPP facilitation milestones, and insurance integration progress as core programme deliverables with defined monitoring indicators and reporting requirements, not optional add-on activities (27).
- **Evidence generation** *[All contexts]:* Commission and fund randomised or quasi-experimental impact evaluations of AT business models in low-income African settings to address the current critical gap in rigorous evidence, with particular priority given to PPP outcome studies, insurance scheme cost-effectiveness analyses, 3D prosthetics lifecycle cost assessments, and given the current scarcity of documented evidence service delivery models specifically adapted to fragile and conflict-affected settings.

#### 7.2.3 Long-term policy and system reforms (beyond 5 years)

- **Systemic programme financing** *[Stable/LMIC]:* Transition from short-cycle, project-based AT support to programme-based systemic investment with five- to ten-year financing commitments aligned with national health sector development plans, enabling the long-horizon investments in workforce, infrastructure, and market development that project funding cycles structurally preclude.
- **Policy-aligned programme support** *[All contexts]:* Increase dedicated programmatic and financial support to projects and programmes that strengthen AT provision in explicit alignment with national rehabilitation policies, strategies, and plans. Donor-funded AT initiatives that operate in parallel to or in ignorance of existing national policy frameworks risk duplicating effort, distorting national health priorities, and creating parallel systems that governments cannot sustain after project closure. All development organisation AT programming should be required to demonstrate alignment with the host country's national rehabilitation strategic plan or equivalent policy instrument as a precondition for funding approval, and to include explicit capacity-strengthening components that increase the government's own ability to finance and deliver AT services within its national system over time (25, 34).

### 7.3 For the private sector

#### 7.3.1 Short-term actions (0 2 years)

- **Technology transfer agreements** *[Stable/LMIC]:* International AT manufacturers should initiate licensing, joint venture, and technology transfer negotiations with African P&O enterprises, prioritising arrangements that build local clinical and manufacturing capacity over direct export models, which perpetuate supply-chain dependence and suppress the development of African AT markets.
- **Sectoral associations** *[Stable/LMIC]:* African AT enterprises should establish or strengthen national and regional sectoral associations to represent the industry collectively in government engagements on procurement policy, insurance benefit package design, and regulatory framework development capabilities that individual enterprises cannot effectively exercise independently.

#### 7.3.2 Medium-term interventions (3 5 years)

- **Dedicated investment vehicles** *[Stable/LMIC]:* The IFC, African Development Bank (AfDB), and Proparco should design and deploy dedicated investment vehicles for AT enterprises, including equity funds, concessional debt instruments, and first-loss guarantee facilities, recognising AT enterprises' direct alignment with Sustainable Development Goal (SDG) 3 (Good Health and Wellbeing), SDG 8 (Decent Work and Economic Growth), and SDG 10 (Reduced Inequalities) (22).
- **Africa-adapted components** *[Stable/LMIC]:* International AT enterprises should partner with African universities, technical institutes, and materials scientists to develop Africa-adapted prosthetic and orthotic components from locally available materials, reducing dependence on imported consumables and generating productive backward linkages into African manufacturing sectors.

#### 7.3.3 Long-term policy and system reforms (beyond 5 years)

- **Vertically integrated value chains** *[Stable/LMIC]:* Develop vertically integrated AT value chains within Africa from raw material processing and component manufacturing through professional clinical fitting, after-sales device service, and maintenance replacing the current near-total import dependence with a self-sustaining continental industry employing certified African professionals.

### 7.4 For the humanitarian community

#### 7.4.1 Short-term actions (0 2 years)

- **Embed market development where feasible** *[Stable/LMIC]:* Embed local market development components in humanitarian AT programming as standard operating practice: local supplier procurement with defined and increasing targets, certified P&O workforce training structured within national regulatory frameworks, and government advocacy for tariff reduction, insurance reform, and quality assurance framework development as budgeted programme activities in every operational context where security and governance conditions permit (31, 32).
- **Localisation standards** *[Stable/LMIC]:* Adopt the ICRC Physical Rehabilitation Programme's progressive localisation model as the sector standard for humanitarian AT procurement, with mandatory, publicly reported supplier diversity targets that systematically increase the share of local and regional suppliers year on year (33).
- **Sustain direct service delivery** *[Fragile]:* In active conflict and chronically fragile settings such as Somalia, Mali, Niger, and the eastern DRC, prioritise the continuity and quality of direct humanitarian AT service delivery itself, recognising that market-building preconditions are not yet present; embed market-development elements opportunistically and incrementally as local security and governance conditions allow, rather than as a fixed programme requirement.

#### 7.4.2 Medium-term interventions (3 5 years)

- **Humanitarian-to-market transition protocols** *[Stable/LMIC]:* Develop and pilot structured transition protocols for the handover of humanitarian AT services to commercial or PPP providers as programming concludes, incorporating transitional financing instruments to support provider establishment during the coverage gap between humanitarian programme exit and commercial viability.
- **Awareness and education platforms** *[All contexts, adapted to local conditions]:* Establish multi-channel community awareness and education platforms combining community radio broadcasting, mobile health applications, structured health worker training on AT prescription and referral, and peer ambassador networks of experienced AT users to systematically reduce information asymmetries at community level and stimulate informed, empowered demand for AT services and devices (11). In insecure areas, radio and community-network channels are likely to remain considerably more reliable than digital or facility-based channels.

#### 7.4.3 Long-term policy and system reforms (beyond 5 years)

- **Institutional advocacy** *[Stable/LMIC]:* Advocate collectively, through existing inter-agency coordination mechanisms, for the mandatory inclusion of AT system development components local procurement, workforce certification pathways, and insurance integration advocacy as standard, funded elements of humanitarian response frameworks, displacing *ad hoc*, project-by-project approaches with systematic and accountable market-building obligations, while preserving flexible, service-first protocols for active conflict settings.

## 8 Terminology note

Precision in AT terminology carries clinical, regulatory, and insurance coverage implications that are practically significant. A prosthesis (plural: prostheses; adjectival form: prosthetic) is an externally applied artificial device that replaces a body segment absent due to congenital deficiency, subsequent surgical amputation, or traumatic loss, most commonly an upper or lower limb or part thereof. An orthosis (plural: orthoses; adjectival form: orthotic) is an externally applied device used to modify the structural and functional characteristics of the neuromusculoskeletal system, typically to support, align, immobilise, prevent or correct skeletal deformity, or redistribute biomechanical loading, without replacing a missing anatomical structure. The terms prosthetics and orthotics refer to the respective professional disciplines and fields of clinical and technical practice, not to individual devices. The distinction between the device noun (prosthesis, orthosis) and the adjectival or disciplinary form (prosthetic, orthotic) is consistently maintained throughout this paper, in accordance with ISPO nomenclature. Where prostheses and orthoses are referenced collectively as a unified product and service category, the abbreviation P&O is used, consistent with established ISPO and WHO usage.

## 9 Conclusion

The account of the patient who travelled two days to a prosthetics clinic and returned home without a prosthetic limb illustrates that the binding constraint on AT access in Africa is not the absence of technology, clinical expertise, or expressed need. It is the absence of the market architecture trade policy, regulatory frameworks, financing systems, and local production capacity required to make reliable, affordable, and sustained AT access structurally possible. That architecture, this paper has argued throughout, cannot be designed identically for every African context; what is structurally possible in Kigali or Nairobi is not equally possible in Mogadishu, Gao, or Goma, and policy prescriptions that ignore this distinction risk irrelevance at best and harm at worst.

The AT access gap in Africa is a structural problem requiring structural solutions: tariff reform and regulatory harmonisation under the AfCFTA; diversified technology transfer drawing on South South, triangular, and North South cooperation; sustainable service delivery models integrating social enterprise, output-based PPPs, and social health insurance; and the systematic inclusion of assistive products in national health financing systems. Humanitarian aid and donations remain essential in emergency and acute transition contexts and, in active conflict settings, are likely to remain the primary mode of AT provision for the foreseeable future but they cannot substitute for market development where the preconditions for such development exist, and should not be designed to attempt to do so. Sustained, equitable, and scalable improvement in AT access requires the deliberate and coordinated design of trade, regulatory, and financing architectures, calibrated explicitly to each country's institutional and security context.

UNIDO Project 240280 in Nigeria represents an early, instructive, and appropriately qualified step toward market transformation. The collaboration among a Japanese social enterprise, a UN development agency, a national regulatory board, and Nigerian national orthopaedic hospitals demonstrates that locally manufactured 3D-printed prosthetic limbs, produced at a fraction of conventional costs by certified Nigerian P&O professionals, are operationally feasible within an African institutional environment possessing the baseline regulatory capacity such a project requires. Long-term clinical viability at scale, lifecycle cost data, and operational sustainability beyond the project cycle remain to be demonstrated through prospective evaluation. The project should be understood as a promising pilot for replication in comparably stable institutional settings, not as a universally transferable model, and not as a proven model even within Nigeria itself.

The comparative evidence from India reinforces the paper's central argument: governments that systematically combine AT manufacturing capacity investment with government-scale demand financing through schemes such as ADIP, innovation ecosystem development, logistics modernisation, and structured public awareness achieve substantially greater and more equitable improvements in AT access than those that rely exclusively on either clinical service expansion or humanitarian donation. Africa can incorporate and adapt these lessons but only with explicit recognition that the continent's institutional and security diversity demands differentiated, not uniform, application.

Decades of field observation from the rehabilitation wards of Bamako and Kinshasa to the orthopaedic hospitals of Kano and Addis Ababa consistently identify the same limiting factor: not a deficit of compassion, clinical skill, or political aspiration, but a deficit of deliberate, context-adapted system architecture. Constructing that architecture through evidence-based trade policy reform, innovative health financing mechanisms, sustainable and contextually adapted business models, structured P&O workforce development, and coherent regulatory frameworks, sequenced according to each setting's institutional and security realities is the **essential, urgent, and as yet unfinished work** of the assistive technology sector in Africa.
